# A Thermo-Active Laccase Isoenzyme From *Trametes trogii* and Its Potential for Dye Decolorization at High Temperature

**DOI:** 10.3389/fmicb.2020.00241

**Published:** 2020-02-19

**Authors:** Xulei Yang, Yuanyuan Wu, Yu Zhang, En Yang, Yuan Qu, Huini Xu, Yuhui Chen, Chagan Irbis, Jinping Yan

**Affiliations:** ^1^Laboratory of Bioconversion, Life Science and Technology College, Kunming University of Science and Technology, Kunming, China; ^2^College of Life Science, Southwest Forest University, Kunming, China

**Keywords:** *Trametes trogii*, thermoactive laccase, thermostable laccase, organic solvent tolerance, dye decolorization

## Abstract

A thermo-activation and thermostable laccase isoenzyme (Lac 37 II) produced by *Trametes trogii* S0301 at 37°C was purified to apparent homogeneity by anionic exchange chromatography and sephadex G-75 chromatography, with 12.3% of yeiled and a specific activity of 343.1 U mg^–1^. The molecular weight of the purified Lac 37 II was estimated to be approximately 56 kDa in 12% sodium dodecyl sulfate polyacrylamide gel electrophoresis (SDS-PAGE). The optimal pH and temperature for the protein was 2.7 and 60°C, respectively. The purified Lac 37 II showed higher resistance to all tested metal ions and organic solvents except for Fe^2+^ and Cd^2+^ at 37°C and the activity of the purified Lac 37 was significantly enhanced by Cu^2+^ at 50 mM. The *K*_*cat*_, *K*_*m*_, and *K*_*cat*_/*K*_*m*_ of Lac 37 II were 2.977 s^–1^, 16.1 μM, and 184.9 s^–1^ μM^–1^, respecively, in the condition of pH 2.7 and 60°C using ABTS as a substrate. Peptide-mass fingerprinting analysis showed that the Lac 37 II matched to the gene-deduced sequences of *lcc3* in *T. trogii* BAFC 463, other than *Lcc1*, *Lcc 2*, and *Lcc 4*. Compared with laccase prepared at 28°C, the onset of thermo-activation of Lac 37 II activity occurred at 30°C with an increase of 10%, and reached its maximum at the temperatures range of 40–60°C with an increase of about 40% of their original activity. Furthermore, Lac 37 II showed the efficient decolorization ability toward triphenylmethane dyes at 60°C, with decolorization rates of 100 and 99.1% for 25 mg L^–1^ malachite and crystal violet in 5 h, respectively, when hydroxybenzotriazole (HBT) was used as a mediator. In conclusion, it is the first time to report a thermo-activation laccase from a thermophilic *T. trogii* strain, which has a better enzyme property and higher decolorization ability among fungal laccases, and it also has a further application prospective in the field of biotechnology.

## Introduction

Laccases (EC1.10.3.2) are a group of copper-containing polyphenol oxidases that are known as “blue enzymes” for green chemistry due to their ability of oxidize diverse substrates which are similar to lignin or the degradation products of major lignin with molecular oxygen as the final electron acceptor. Due to their high catalytic efficiency and broad substrate specificity, laccases are used in various fields, including biopulping, delignification, biobleaching, environmental pollutants bioremediation, dye decolorization, etc. (Riva, 2006; Bertrand et al., 2017; Younes et al., 2019).

Fungi, especially white rot fungi, are the main laccase producers in nature, and the potential laccase producing strains be utilized in industrial application (Bertrand et al., 2017). Until now, many laccase-producing fungi have been studied and most of the fungi can produce several laccase isozymes (usually more than 10 isoenzymes in the same fungus strain) that showed different kinetic and physicochemical features, which makes it possible to seek new laccase isoenzymes and meets the demands in the industrial applications (Janusz et al., 2013; Zhuo et al., 2016; Zheng et al., 2017). However, the expression of different laccase isoenzymes in the same strain depends on many factors such as the presence of inducers (especially Cu^2+^ and phenolic compounds), the ratio of carbon and nitrogen, age of the culture and heat shock treatment (Baldrian, 2006; Piscitelli et al., 2011; Janusz et al., 2013; Zhuo et al., 2016; Bertrand et al., 2017). Untill now, few laccase isoenzymes have been isolated and characterized, usually one or two isoenzymes per fungus strain, and most of the laccases isoenzymes isolated so far are found sensitive to extreme conditions of temperature, pH, metal ions, etc. (Janusz et al., 2013; Fonseca et al., 2015; Jaiswal et al., 2015; Othman et al., 2018).

Laccase isoenzymes of thermophilic bacteria and fungi usually possess many attractive properties including high thermal stability, thermo-activation (stimulation of enzyme activity by pre-incubation), and tolerance to organic solvents and ionic concentrations (Hildén et al., 2007; Younes and Sayadi, 2011; Yan et al., 2014a, b), which are demanded biobleaching of pulp and treatment of colored industrial effluents (Wong et al., 2000; Asgher et al., 2008). Previous reports have found that increasing the temperature for laccase production (Tong et al., 2007) and heat shock treatment (Wang et al., 2012) in *Trametes* strains can induce the expression of different laccase isoenzymes and enhance laccase activity. In our experiment, we observed that *T. trogii* S0301 strain can grow at 37°C, but to date, only two native laccase isoenzymes (named Lcc1 and Lcc2) have been purified from this strain, and both of them were obtained from the supernatants cultured at 28°C (Colao et al., 2003; Yan et al., 2014a).

Thermo-active enzymes usually are more thermotolerant (Rathi et al., 2000; Hildén et al., 2007; Younes and Sayadi, 2011; Campos et al., 2016). Although the first thermo-active laccase was isolated as early as 1993, few thermo-active laccase isoenzymes have been isolated until now, such as laccase isoenzymes from *Fomes sclerodermeus*, *T. hirsutus*, *Coliolus zonatu*, *Marasmius quercophilus*, *Myceliophtora thermophile*, and *Scytalidium thermophilum* (Hildén et al., 2007; Younes and Sayadi, 2011). Thermo-activation has been observed in the heterogeneous expressed LCC3 of *T. trogii* BAFC 463 in *Pichia pastoris* (named the recombined LCC3) (Campos et al., 2016), but there are no reports about the thermo-active laccase isoenzyme originated from *Trametes* until now.

To explore the potential application of laccases in the thermotolerant *T. trogii* S0301 strain, laccase was obtained from this strain cultured at the temperature of 37°C. The main objectives of current study were (i) to purify and identify the laccase of *T. trogii* S0301 produced at 37°C; (ii) to characterize this laccase isoenzyme; and (iii) to assess the potential application of this laccase isoenzyme by dye decolorization experiments.

## Materials and Methods

### Chemicals and Strain

2,2′-Azino bis (3-ethylbenzothiazoline-6-sulfonic acid) (ABTS) and dyes (malachite green, bromophenol blue, and crystal violet) were purchased from Sigma-Aldrich and Merck, respectively. *T. trogii* S0301 strain employed in the present study was stored in the strain collection of Laboratory of Bioconversion of Life Science and Technology College, Kunming University of Science and Technology, and maintained on a GYP slant at 4°C (Yan et al., 2014a, b).

### Laccase Production

Four 1-cm^2^ plugs of the GYP plates incubated at 28°C for 4 days were excised with a sterilized cutter and added to each 250 mL Erlenmeyer flask containing 50 mL of GYP. After another 5 days incubation, the mycelia were homogenized with glass beads (0.3 mm in diameter) and transferred to GYP broth containing 2 mM Cu^2+^ with 10% (v/v) of the seed culture broths. The cultures were incubated in a rotary shaker at 200 rpm at 28 and 37°C, respectively. Ten-day-old liquid cultures were obtained by centrifuging (8000 rpm, 15 min) and the cell-free supernatants were designated as the crude enzyme for the further study.

### Laccase Activity and Laccase Thermo-Activation Analysis

Laccase activity was determined with ABTS as the substrate. The 1.5 mL substrate solution includes 2 mM ABTS, 100 mM phosphate citrate buffer (pH 4.0), and 0.1 mL appropriately diluted crude or purified enzyme was used to determine the activity. The increase in absorbance was monitored at 420 nm for 3 min. One unit of the enzyme activity was defined as the amount of the enzyme that oxidized 1 μmol of the ABTS per minute according to the methods described by Yan et al. (2014b).

For thermo-activation analysis, the enzymes were pretreated at different temperatures (30–80°C) for 30 min in 100 mM phosphate citrate buffer (pH 4.0), and then thoroughly cooled on ice for another 30 min. Next, the residual laccase activity was determined. The same amount of enzymes that were not heat-treated but placed on ice as positive control, and the heat-denatured enzymes were served as the negative control. All the assays were carried out in triplicate.

### Laccase Purification

The purification of the laccase from the crude enzyme of 10-day-old liquid cultures under 37°C with the addition of 2 mM Cu^2+^ was carried out as described in Yang et al. (2011). Briefly, the total protein was precipitated from the crude enzyme using ammonium sulfate (80% saturation). After dialysis, the enzyme solution was successively treated with a Q Sepharose^TM^ ion-exchange chromatography column (GE Healthcare) and a Sephadex G-75 Medium chromatography (Biotopped) column. The fraction containing laccase activity was collected and stored at −20°C for further studies. Sodium dodecyl sulfate-polyacrylamide gel electrophoresis (SDS-PAGE) and native sodium dodecyl sulfate-polyacrylamide gel electrophoresis (Native-PAGE) were carried out according to Shi et al. (2014).

### Laccase Identification

The purified laccase was further separated by Native-PAGE. After the electrophoresis, the gel was stained with citrate-phosphate buffer (100 mM, pH 4.0) containing 1.0 mM ABTS and the laccase band was collected for further study. MALDI-TOF/TOF-MS analysis used commercial service provided by Sangon Biotech on 4800 Plus MALDI TOF/TOFTM Analyzer (ABI, Foster City, CA, United States). Mass spectra were obtained in positive ions regime using reflectron. The program Mascot^1^ was used for protein identification by “peptides fingerprints” and fragmentation spectra. The database NCBI was used for searching homology among proteins of all organisms and fungi with the accuracy mentioned taking into account possible methionine oxidation by atmospheric oxygen and possible modification of cysteine by acrylamide (Younes and Sayadi, 2011; Zheng et al., 2017).

### pH and Temperature Effects on the Purified Laccase and Kinetic Analysis

#### Effects of pH on Laccase Activity and Laccase Stability

Characterization analysis of the purified laccase was carried out according to Yan et al. (2014b). To determine the optimum pH of the purified laccase, the laccase activity was assayed in 100 mM citrate-phosphate buffer adjusted to various pH values between 2.0 and 8.0, with 1.0 intervals at 30°C. Effect of pH on the stability of the purified laccase was studied by verifying the remaining activity after incubating the purified enzyme in the buffer solutions mentioned above at 30°C for 36 h. Next, the residual laccase activity of each treatment was compared with the control under the standard assay conditions. All the experiments were performed in triplicate.

#### Effects of Temperature on Laccase Activity and Laccase Stability

To determine the optimum pH of the purified laccase, enzymatic reaction was conducted at temperatures from 30 to 80°C with the enzyme in 100 mM citrate-phosphate buffer (pH 4.0), with 10°C intervals (Wu et al., 2010). For the thermostability analysis, the half-life at certain temperature (*T*_1__/__2_) was determined after the purified laccase incubated at given temperature (60, 70, or 75°C) in phosphate citrate buffer (100 mM, pH 4.0) with different time intervals. The residual laccase activity was determined by the standard conditions and the activity of untreated enzyme was used to represent 100% relative activity. All the experiments were performed in triplicate.

#### Effects of Metal Ions on Laccase Activity

Metal ions including Na^+^, Fe^2+^, Cd^2+^, Mn^2+^, Zn^2+^, Mg^2+^, Co^2+^, and Cu^2+^ were added to the standard laccase reaction mixture with a final concentration of 5 and 100 mM, and the residual activities were measured under the standard conditions (Shi et al., 2014). The laccase activity of the reaction mixture without metal ions was recorded as 100%. All the experiments were performed in triplicate.

#### Effects of Various Organic Solvents on Laccase Activity

To determine the effects of organic solvents on laccase activity, commonly used solvents including methanol, ethanol, and acetonitrile were added to the standard laccase reaction mixture with a final concentration of 1, 5, or 10% (v/v) and the residual activities were measured under the standard conditions (Shi et al., 2014). The laccase activity of the reaction mixture without organic solvent was recorded as 100%. All the experiments were performed in triplicate.

#### Kinetic Study

The kinetic constants (*K*_*m*_ and *K*_*cat*_) were determined by using ABTS as substrate in series concentrations ranged from 0 to 2 mM at the optimal condition of the purified laccase in 100 mM citrate–phosphate buffer (pH 2.7) at 60°C. The laccase activity for each substrate concentration was determined three times. The *K*_*m*_ and *K*_*cat*_ values were evaluated by the Lineweaver–Burk plot using the Originpro 8 for Windows. All the experiments were performed in triplicate.

### Dye Decolorization

Decolorization experiments were carried out according to Yan et al. (2014a, b). The dye decolorization reaction was conducted at 60°C with or without the addition of hydroxybenzotriazole (HBT) as a laccase mediator (Younes et al., 2007). Decolorization efficiency was calculated according to the following formula: decolorization (%) = *A*_0_−*A*_*t*_/*A*_0_^∗^ 100%, where *A*_0_ is the initial absorbance and *A*_*t*_ is the final absorbance (Younes et al., 2016).

### Statistical Analysis

All data were presented as mean ± standard deviation (SD) for three replications for each sample. The ANOVA test using the software of Origin pro 8 for Windows used to analyze the experiment data. *P*-value < 0.05 was considered significant. In addition, all statistical charts were drawn by Origin pro 8 for Windows. Protein sequence alignment was analyzed by DNAMAN software.

## Results and Discussion

### Thermo-Activation Comparison of the Crude Enzymes From *T. trogii* S0301 Produced at 28 and 37°C

**FIGURE 1:**
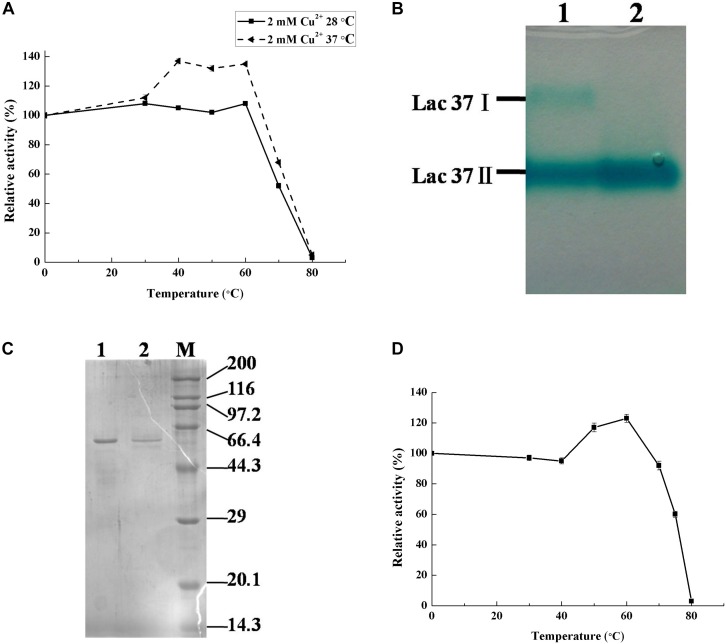
Thermo-activated analysis and purification of laccase from *T. trogii* S0301. Native-page **(B)** and SDS-page **(C)** analysis. Lanes 1 and 2 are the crude and purified laccase produced at 37°C, respectively. The crude laccase activity of the culture filtrate produced at 37 and 28°C were 3330.62 and 4861.11 U L^–1^ **(A)**. Thermo-activated analysis of purified Lac 37 II from 37°C, which the original activity is 343.06 U mg^–1^ **(D)**. Activity is represented as % relative to the not heat-treated, assigned as 100%. Data points are the average of triplicate measurements, and the error bars represent the standard deviation.

Previously, the thermo-active laccase isoenzymes have been observed in the thermophilic or thermotolerant strains, such as *Melanocarpus albomyces*, *M. thermophila*, and *S. thermophilum* (Kiiskinen et al., 2002; Younes and Sayadi, 2011). The original crude laccase activity of the culture filtrate produced at 37 and 28°C were 3330.62 and 4861.11 U L^–1^, respectively. We observed that the crude laccase prepared at 37°C showed temperature-dependent activation after 30 min of thermal treatment under the different temperature conditions, while the crude laccase of this strain prepared at 28°C did not show obvious thermo-activation under the same experiment conditions (Figure 1A). The onset of thermo-activation of laccase activity occurred at 30°C with an increase of 10%, and reached its maximum at the temperatures range of 40–60°C with an increase of about 40% of their original activity (Figure 1A). Further raising temperatures caused the loss of the enzyme activity and the disappearance of thermo-activation. Until now, only two laccase isoenzymes (Lcc1 and Lcc2) have been purified from *T. trogii* strains and neither of them is thermo-active (Yan et al., 2014a; Campos et al., 2016). To further explore whether thermo-activation due to different laccase isoenzymes in the crude enzyme, laccase of this strain produced at 37°C was purified and identified.

### Laccase Purification

Using Native-PAGE, two main laccase isoenzymes (Lac 37 I and Lac 37 II) were detected from the crude laccase of *T. trogii* S0301 which produced at 37°C in liquid GYP medium added with 2 mM Cu^2+^ (Figure 1B). Laccase which secreted in culture medium was purified after ammonium sulfate precipitation. By the first step of the purification, the specific lacase activity of crude laccase was changed from 55.3 to 96.0 U mg^–1^ (Table 1), followed by the anionic exchange chromatography column and the Sephadex G-75 chromatography column kept in tandem. Six protein peaks were obtained by anionic exchange chromatography column, and the first peak showed the laccase activity (111.9 U mg^–1^) (Table 1). The protein solution from peak 1 was collected and then purified by Sephadex G-75 chromatography column. A final specific activity of 343.06 U mg^–1^ was achieved. A purification fold of 6.2 and a total enzyme yield of 9.6% were obtained (Table 1). A single laccase band detected on Native-PAGE indicated that only Lac 37 II was purified in this study. Lac 37 II showed a single band by SDS-PAGE, with a predicted molecular mass of approximately about 60 kDa (Figure 1C). Most studies showed that the molecular weight of fungal laccase monomer is between 50 and 100 kDa, which is consistent with the molecular weight of the purified Lac 37 II (Kunamneni et al., 2008). However, we failed to obtain the other laccase isoenzyme, Lac 37 I, in this study. In order to obtain the Lac 37 I, maybe we should change the purification conditions.

**TABLE 1:** Summary of laccase purification from *T. trogii* S0301.

Purificatin step	Total volume (mg)	Total activity (U)	Total protein (mg)	Specific activity (U mg^–1^)	Yield (%)	Purification fold
Crude laccase	1712	3330.62	59.6	55.31	100	1
Ammonium sulfate precipitation	59.57	735.03	11.137	95.99	22.07	1.74
Anionic exchange chromatography	4.83	440.5	4.3	111.91	13.22	2.02
Sephadex G-75 chromatography	3.4	321	1.02	343.06	9.6	6.2

The purified Lac 37 II exhibited obvious temperature-dependent activation at temperatures from 40 to 60°C and reached its maximum at 60°C, with an increase of approximately 45% of the original activity (343.06 U mg^–1^) (Figure 1D), which was similar to that of the crude enzyme of *T. trogii* S0301 produced at 37°C (Figure 1A). Thus, we suggest that Lac 37 II is the main source of the thermo-active laccase. In our study, the thermo-activation temperature range was agreed with other thermo-cativation laccase isoenzymes, but a highest increasing rate of laccase activity was observed in Lac 37 II, which have approximately 20% of increase compared to that of *Physisporinus rivulosus* and *S. thermophilum* (Hildén et al., 2007; Younes and Sayadi, 2011).

### pH and Temperature Effects on Lac 37 II and Kinetic Analysis

According to the literature, the enzyme properties of many purified and recombined laccases mainly from *T. trogii* strains and some thermotolerant fungi are summarized in Table 2.

**TABLE 2:** Comparison of kinetic properties of the purified laccases mainly from strains of *Trametes* genus.

Strain	Specific activity (U mg^–1^)	*K*_*m*_ (μM)	*K*_*cat*_ (s^–1^)	*K*_*cat*_/*K*_*m*_ (s^–1^ μM^–1^)	Optimal condition (Tem/pH)	*T*_1/2_ (min)	References
*T. trogii* S0301 at 37°C (Lac 37 II)	343.06	16.1	2977	184.96	60°C/2.7	>360 (at 60°C) 120 (at 70°C)	This study
*T. trogii* S0301 at 28°C	352.1	69	7958	115	45°C/3.0	180 (at 60°C)	Yan et al., 2014a
*T. trogii* BAFC 463^a^	–	–	–	–	50°C/4.4	>120 (at 60°C) <60 (at 70°C)	Grassi et al., 2011
*T. trogii* 201	152	30	3.3	0.11	−/3–3.5	–	Garzillo et al., 1998
*T. trogii* YDHSD		7.32	260	35.6	70°C/2.2	90 (at 60°C)	Ai et al., 2015
Recombined Lcc 1 of *T. trogii*	232	9.2	98.1	10.6	−/2.2	Lost 90% after 3 h at 60°C, pH 6	Colao et al., 2006
Recombined Lcc 2 of *T. trogii*	–	218	5.8	0.03	−/2.5	–	Colao et al., 2009
Recombined Lcc 3 of *T. trogii* BAFC 463	–	250	399	1.59	50°C/2.7	>180 (at 60°C) 45 (at 70°C)	Campos et al., 2016
*T. trogi* LacA	11.85	54.6			50°C/4.5	>240 (at 40°C) Unstable at 60°C	Guan et al., 2011
*T. trogi* LacB	4.52	17.7			60°C/4.0		
*T. pubescens*	18.543	105	876	8.34	50°C/5.0	120 (at 75°C)	Si et al., 2013
*T. versicolor* sdu-4	1320	47.5	284	99.7	−/2.2	132 (at 70°C)	Zhu et al., 2011
*S. thermophilum* at 42°C	139.4	260	1431	5.5	80°C/5.0	120 (at 65°C) 90 (at 70°C)	Younes et al., 2007
*Cladosporium cladosporioides* at 42°C	–	19.6	–	–	40–70°C/3.5	5 (at 70°C)	Halaburgi et al., 2011
*Echinodontium taxodii 2538*		41.4	–	–	60°C/3	>2 (at 50°C)	Shi et al., 2014

*^*a*^Laccase was purified but the data were based on the crude laccase. All data in this table were using ABTS as substrate.*

**FIGURE 2:**
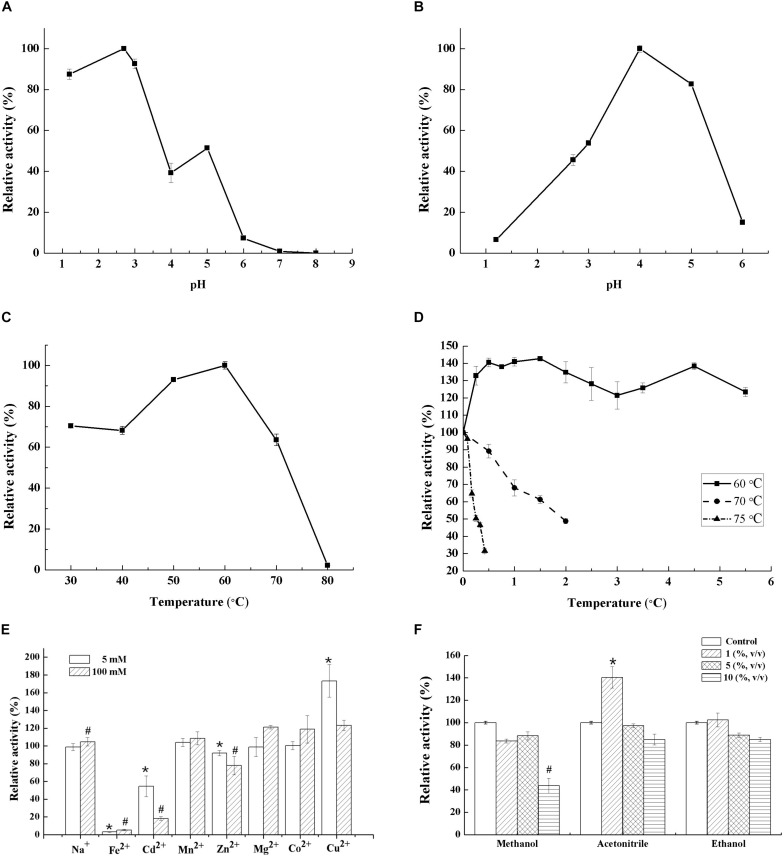
Characterization of Lac 37 II. Effect of pH on laccase activity **(A)** and stability for 36 h **(B)**. Effect of temperature on laccase activity **(C)** and stability **(D)**. Effect of metal ions (5 or 100 mM) and organic solvent on laccase activity **(E,F)**. Laccase activity that without heat treatment, metal ions, and organic solvent at pH 4.8 was 343.06 U/mg. Activity is represented as % relative to laccase activity under the standard conditions, assigned as 100% for stability studies. The maximum laccase activity was recorded as 100% for the optimal temperature and pH studies. The data are presented as means from three independent measurements ± the standard deviations (indicated by the error bars). *p and *^#^p* < 0.05, as determined by one-way ANOVA.

Generally, fungal laccases demonstrate their optimal pH of 2.0–6.0 using ABTS as substrate (Si et al., 2013; Shi et al., 2014; Ai et al., 2015). Lac 37 II, in this study, exhibited maximal activity at pH 2.7, which is in accordance with the recombined LCC3 and the purified laccases from *T. trogii* S0301 produced at 28°C with the optimum of pH 2.7 and 3.0 using ABTS as the substrate, respectively (Figure 2A and Table 2; Yan et al., 2014a; Campos et al., 2016). The original activity of Lac 37 II was stable, maintaining >80%, after incubation at pH 4 and 5 for 36 h (Figure 2B). When the pH was <3, the laccase activity was significantly inhibited, with an activity of 18%. These results are in accordance with other fungal laccases (Shi et al., 2014; Yan et al., 2014b).

The optimum temperature for Lac 37 II was 60°C with ABTS as a substrate, which was higher than that of the recombined LCC3 with the highest laccase activity at 50°C (Figure 2C and Table 2). The half-lives of enzymatic activity at various temperatures (*T*_1__/__2_) at pH 4.0 were >12 h at 60°C, 2 h at 70°C, and 15 min at 75°C with ABTS as the substrate (Figure 2D). Based on the thermostability assays, Lac 37 II in this study exhibited a notable advantage over almost all laccases from the *Trametes* genus and other sources (Table 2), except for that of *T. versicolor* sdu-4 (*T*_1__/__2_ of 132 min at 70°C) (Zhu et al., 2011) and *T. pubescens* (*T*_1__/__2_ of 120 min at 75°C) (Si et al., 2013).

Kinetic analysis was carried out with ABTS as a substrate at optimal conditions (pH 2.7 and 60°C). The *K*_*m*_, *K*_*cat*_, and *K*_*cat*_/*K*_*m*_ of Lac 37 II were 16.1 μM, 2977.8 s^–1^, and 184.9 s^–1^ μM^–1^, respectively (Table 2). Lac 37 II in this study possessed higher thermostability and catalytic efficiency, which makes the laccase isoenzyme have further prospective for the biotechnological applications.

### Effect of Metal Ions on Activity of Lac 37 II

Metal ions are widely distributed in environmental pollutants, and laccases with higher resistance to metal ions are thus attractive (Younes and Sayadi, 2011). In this study, the effects of several metal ions (Na^+^, Fe^2+^, Cd^2+^, Mn^2+^ Zn^2+^, Mg^2+^, Co^2+^, or Cu^2+^) on Lac 37 II were investigated. The purified Lac 37 II exhibited a high degree of resistance to some metal ions. When the concentration of metal ions was 5 and even 100 mM, Na^+^, Mn^2+^, Mg^2+^, Co^2+^, and Cu^2+^ had little effects on the laccase activity. However, the other metal ions such as Fe^2+^, Cd^2+^, and Zn^2+^ showed inhibitory effects on the activity of Lac 37 II, especially Fe^2+^. Fe^2+^ completely inhibited the activity of Lac 37 II even at a low concentration (5 mM), and laccase activity decreased to 54.5 and 90.2% in the presence of Cd^2+^ and Zn^2+^ at 5 mM, respectively (Figure 2E). Some reports have shown that metal ions have some effects on laccase, most of which inhibit laccase activity (Hu et al., 2014; Zhuo et al., 2015). Previous studies have demonstrated that even at low concentrations, Fe^2+^ (1 or 10 mM) can strongly inhibit laccase activity in many strains, including *Pleurotus ferulae*, *Pycnoporus* sp., *T. trogii* YDHSD, *S. thermophilum*, *Trametes* sp. MA-X01, and *T. trogii* S0301 (Younes and Sayadi, 2011; Yan et al., 2014a; Ai et al., 2015; Wang et al., 2018). And the purified rLAC-EN3-1 from *P. pastoris* was also sensitive to Cd^2+^ with relative activities of 62% at 10 mM and 18% at 100 mM (Zhuo et al., 2015).

Cu^2+^, by contrast, obviously enhanced the laccase activity of Lac 37 II with relative activities of 173.3 and 123.1% at 5 and 100 mM, respectively (Figure 2E), which could be due to the role of free copper ions as reducing agents in the solution and reducing the copper center in laccase (Qiao et al., 2017). Those results were in good agreement with the laccase of this strain produced at 28°C and of *T. pubescens* with a relative activity of 128% at 100 mM Cu^2+^ and 111.3% at 25 mM Cu^2+^, respectively (Si et al., 2013; Yan et al., 2014b). Similarly, there was activation of laccase by 10 mM Cu^2+^ from *Sporothrix carnis* CPF-05. However, the laccases of *Bacillus subtilis* cjp3 and *T. trogii* YDHSD were sensitive to Cu^2+^ with relative activities of 14% (Qiao et al., 2017) and 80.9% at 10 mM (Ai et al., 2015), respectively. Younes and Sayadi (2011) reported that 100 mM Co^2+^ greatly inhibited laccase activity in *S. thermophilum* and *F. fomentarius*, compared with relative activities of 100.3 and 77.8% at 100 mM for laccases from *T. trogii* S0301 at 37 and 28°C, respectively.

### Effect of Organic Solvents on Activity of Lac 37 II

Many substrates of laccases are organic pollutants that contain high concentrations of organic solvents used to enhance solubility (Maté et al., 2010). These will lead to undesirable side reactions of hydrolysis, which is not conducive to thermodynamic equilibrium and difficult to product recovery. The reaction of enzyme catalyst in various organic solvents is greatly limited (Klibanov, 2001). Similarly, the existence of organic solvents is also involved in the application of enzyme membrane immobilization, although it ensures the stability of enzyme to a certain extent (Liu et al., 2019). Thus, fungal laccases with organic cosolvent tolerance have practical uses. The effects of three common solvents (methanol, ethanol, and acetonitrile at a concentration of 1, 5, and 10%) on Lac 37 II activity was investigated. The purified Lac 37 II maintained >80% of its activity in buffer containing ethanol, even at high concentration (10%, v/v). Among all tested organic solvents, 1% (v/v) acetonitrile increased laccase activity by approximately 40.4%, while activity slightly declined to 97.3 and 85.0% of the control at 5 and 10% (v/v), respectively (Figure 2F). The tolerance of Lac 37 II to acetonitrile and ethanol in this study were similar to that of laccase-like enzyme from the marine sediment samples (Yang et al., 2018). The promotive effect of acetonitrile on laccase activity has been confirmed for the crude laccase of *T. trogii* LK13 (Yan et al., 2015). In addition, methanol at concentrations ranging from 1 to 5% (v/v) slightly lowered activity by 11.7–16.3%, and 10% ethanol (v/v) led to a 56.1% loss of activity. Similarly, the catalytic activity of laccase in *S. carnis* CPF-05 was almost lost when 10% of the organic solvents added (Olajuyigbe and Fatokun, 2017). In addition, the solvent tolerance of the enzyme is considered to be positively correlated with the thermal stability, which is also in line with the thermo-active and solvent tolerance of Lac 37 II in this study (Rasekh et al., 2014).

### Laccase Identification

**FIGURE 3:**
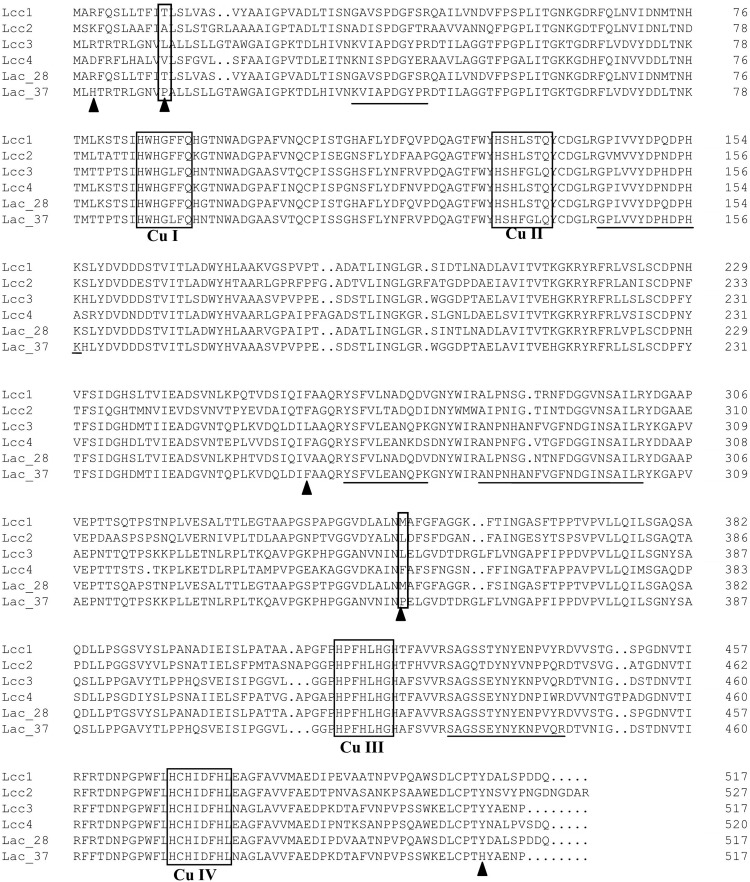
Multiple amino acid sequences alignments of Lac 37 II with other fungal laccases of *T. trogii*. Four copper-binding conserved domains of typical laccase: CuI (HWHGFFQ), CuII (HSHLSTQ), CuIII (HPFHLHG), and CuIV (HCHIDFHL) were boxed in black. The underline showed the internal peptide sequences of Lac 37 II based on the results of MALDI-TOF MS. Lac_28 and Lac_37 were the purified laccase of *T. trogii* S0301 strain produced at 28°C and Lac 37 II at 37°C, respectively. ▲ indicated the five different amino acids in the gene-deduced sequences between Lac 37 II and Lcc 3.

Using MALDI-TOF MS, five peptides of Lac 37 II were obtained and the sequences of them were dertermined as follows: KVIAPDGYPR, GPLVVYDPHDPHK, YSFVLEANQPK, ANPNHANFVGFNDGINSAILR, and SAGSSEYNYKNPVQR. These peptides from Lac 37 II accurately matched to the gene-deduced sequences of *lcc3* (GenBank KU055623) in *T. trogii* BAFC 463, but did not match other laccase isoenzymes of *T. trogii* strains (Lcc1, Lcc 2, Lcc 4, or the purified laccase of *T. trogii* S0301 at 28°C) (Figure 3). The theoretical protein molecular weight was 56 kDa, which is similar to the predicted molecular weight by SDS-PAGE. Previously, *lcc3* of *T. trogii* BAFC 463 has been expressed in *P. pastoris*, and the recombined LCC3 showed excellent thermostability and thermo-activation (Campos et al., 2016). The LCC3 was assumed to be due to the thermal stability observed in *T. trogii* BAFC 463 culture filtrates, but in their study, LCC3 did not purified from the fermentation supernate (Campos et al., 2016). Based on the results of laccase identification, we speculated that Lac 37 II purified in our study is the native LCC3, and it was the third laccase isoenzyme isolated from *T. trogii*.

We also observed that Lac 37 II showed certain great advantages over the recombined Lcc 3, such as smaller molecular weight and higher thermostability (Figure 1). In our study, Lac 37 II showed a smaller molecular mass than that of the recombined LCC3 in *T. trogii* BAFC 463. In addition, the optimum temperature, thermostability, and decolorizing efficiency of Lac 37 II were higher than those of the recombined LCC3 (Table 2).

The possible reasons for those differences are the post-translational modifications in different hosts (yeasts and *T. trogii*), especially the glycosylation (Maestre-Reyna et al., 2015). Previous studies have proved that the recombined laccases in *P. pastoris* were always hyperglycosylated along with the changes of molecular mass and enzymatic properties, and the mechnism is that the glycosylation profile acts as the regulatory modules for substrate binding and turnover (Younes et al., 2007; Odón et al., 2009; Neha et al., 2012; Maestre-Reyna et al., 2015; Peter, 2016). In addition, we also found that the two amino acids (P13 and P351) of Lac37 II are different from lac3 (L13 and L351) (Figure 3). By predicting the protein structure of Lac 37 II, it was found that two prolines were located in the loop region. Suzuki et al. (1987) and Watanabe et al. (1991) considered that the proline may improve the thermal stability of protein by reducing the skeleton entropy of protein unfolding in the proper β-corner or random curl position. Two mutant lipases improved thermal stability by proline substitution mutagenesis, which were more stable than wild type (Mohammadi et al., 2016). Therefore, the difference of proline may be another factor that affects the thermal stability of Lac 37 II.

### Dye Decolorization

The crude and purified laccase of *T. trogii* S0301 produced at 28°C and the recombined LCC3 all showed high efficiency toward decolorization of triphenylmethane dyes (Grassi et al., 2011; Yan et al., 2014a, b; Campos et al., 2016). Based on these results, two triphenylmethane dyes (malachite green and crystal violet) were chosen in this study to assess the potential application of this laccase isoenzyme. Due to the reasonably good thermostability of the purified laccase, dye decolorization analysis was carried out at 60°C in this study.

Without the addition of the laccase mediator, Lac 37 II was less effective against malachite green, and crystal violet, with maximum decolorization of 8.6% for 25 mg L^–1^ crystal violet and 16.0% for 10 mg L^–1^ malachite green in 15 h, which was similar to the recombined LCC3 (Campos et al., 2016), but much lower than those of the purified laccase from *T. trogii* S0301 produced at 28°C with the maximum decolorization of 95.7% in 11 h for all dyes at the same concentration (Yan et al., 2014a) (Table 3).

**TABLE 3:** Comparison of the decolorization ability of Lac 37 II with other *Trametes* laccases.

Strains	Laccase activity (U mL^–1^) ^a^	Dyes (mg L^–1^)^b^	Condition	Time (h)	Decolorization rate (%)	Laccase mediators^c^	References
*T. trogii* S0301 at 37°C	0.25	MG (10)	pH 4.0 at 60°C	1	100	+	This study
		MG (10)		20	16	−	
		MG (25)		3	100	+	
		CV (25)		5	100	+	
		CV (25)		20	8.6	−	
Recombined Lcc 3 of *T. trogii* BAFC 463	1	MG (18)	pH 4.5 at 60°C	24	0	−	Campos et al., 2016
		MG (18)		2	82.8	+	
*T. trogii* BAFC 463	6.5	MG (8)		24	98	+	Grassi et al., 2011
		MG (8)		24	25	−	
*T. trogii* S0301 at 28°C	0.25	MG (10)	pH 4.0 at 28°C	11	83.6	−	Yan et al., 2014a
		CV (25)		11	95.7	−	

*^*a*^Laccase (U mL^–1^) indicated the final laccase activity in the reaction mixture. ^*b*^MG (malachite green), CV (crystal violet). ^*c*^+/− indicated with or without the laccase mediators.*

**FIGURE 4:**
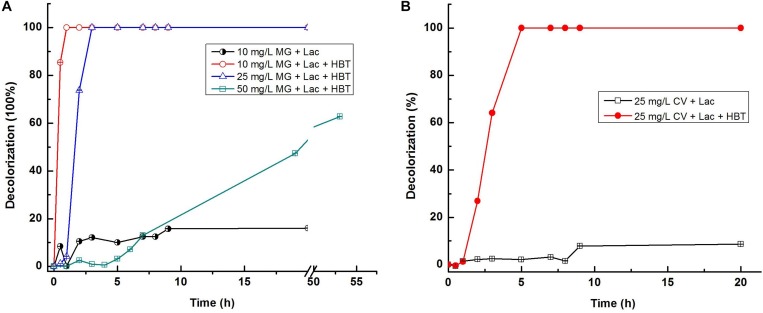
Dye decolorization by Lac 37 II. Reactions were carried out using citrate-phosphate buffer (100 mM, pH 6.0) at 60°C. MG (malachite green) and CV (crystal violet). The degradation of malachite green **(A)** and crystal violet **(B)** were carried out using citrate-phosphate buffer (100 mM, pH 6.0) at 60°C. Each value is the mean value ± standard error of the mean of triplicates. Each value is the mean value ± standard error of the mean of triplicates.

Previous studies have indicated that the natural or artificial laccase mediators can increase decolorization of dyes by both the purified and crude laccases from many strains, including *T. trogii* and *T. villosa* (Grassi et al., 2011; Campos et al., 2016). To confirm whether laccase mediators can improve the decolorization ability of Lac 37 II, 1-HBT was chosed as a laccase mediator. When HBT was added at the concentration of 2 mM, the highest decolorization rate of Lac 37 II was detected as approximately 100% for 10 mg L^–1^ malachite green within 1 h (Figure 4A). Lac 37 II efficiently decolorized 25 mg L^–1^ malachite green, with maximum decolorization of 73.7 and 99.1% in 2 and 3 h, respectively (Figure 4A). To explore the decolorization ability of Lac 37 II at elevated concentrations of malachite green, 50 mg L^–1^ malachite green was used. Lac 37 II was able to decolorize with maximum decolorization of 47.3 and 62.7% in 20 and 53 h, respectively (Figure 4A). HBT also greatly enhanced the decolorization efficiency of Lac 37 II for crystal violet, with decolorization of 26.9, 64.1, and 99.1% in 2, 3, and 5 h, respectively, while Lac 37 II without HBT showed almost no effect on crystal violet even after 20 h of incubation, with decolorization of 9.1% at 20 h (Figure 4B).

In this study, HBT was chosen as the sole laccase mediator, and a greater increase in decolorization rate was obtained for all tested dyes. However, HBT has been demonstrated as a laccase mediator with a slight or no effect on dyes decolorization (Canas and Camarero, 2010; Campos et al., 2016). Thus, laccase mediators, especially more natural and effective ones, can be optimized in future studies to enhance the decolorization efficiency of Lac 37 II.

## Conclusion

In this study, Lac 37 II, a novel native laccase isoenzyme of *Trametes trogii* S0301 was obtained by incubating this strain at 37°C, which is higher than the normal cultivation temperature of fungi. By purification and identification, we found that Lac 37 II is the third native laccase isoenzyme from *T. trogii* strains, and it is also the first thermo-active and the more thermostable isoenzyme of *Trametes* genus strains. With higher thermostability and catalytic efficiency, this laccase isoenzyme can efficiently decolorize triphenylmethane dyes with the addition of a laccase mediator, which makes Lac 37 II have further prospective for biotechnological applications.

## Data Availability Statement

All datasets generated for this study are included in the article/supplementary material.

## Author Contributions

XY has carried out enzyme activity determination, thermal stability analysis, and laccase purification. YW was responsible for the effect of temperature and pH on the activity and stability of laccase. YZ was responsible for the effect of organic dissolution and metal ions on laccase activity. EY was responsible for the identification of laccase. YQ was responsible for the decolorization of dyes by laccase. HX was responsible for SDS-PAGE and Native-PAGE. YC was responsible for the preparation of laccase. CI has revised the original manuscript. JY has conceived the experiment plan, supervised the experiment process, and wrote the original manuscript.

## Conflict of Interest

The authors declare that the research was conducted in the absence of any commercial or financial relationships that could be construed as a potential conflict of interest.

## Footnotes

1 http://www.matrixscience.com
